# Dopamine Receptor Antagonists as New Mode-of-Action Insecticide Leads for Control of *Aedes* and *Culex* Mosquito Vectors

**DOI:** 10.1371/journal.pntd.0003515

**Published:** 2015-03-20

**Authors:** Andrew B. Nuss, Karin F. K. Ejendal, Trevor B. Doyle, Jason M. Meyer, Emma G. Lang, Val J. Watts, Catherine A. Hill

**Affiliations:** 1 Department of Entomology, Purdue University, West Lafayette, Indiana, United States of America; 2 Department of Medicinal Chemistry and Molecular Pharmacology, Purdue University, West Lafayette, Indiana, United States of America; Liverpool School of Tropical Medicine, UNITED KINGDOM

## Abstract

**Background:**

New mode-of-action insecticides are sought to provide continued control of pesticide resistant arthropod vectors of neglected tropical diseases (NTDs). We previously identified antagonists of the AaDOP2 D1-like dopamine receptor (DAR) from the yellow fever mosquito, *Aedes aegypti*, with toxicity to *Ae*. *aegypti* larvae as leads for novel insecticides. To extend DAR-based insecticide discovery, we evaluated the molecular and pharmacological characteristics of an orthologous DAR target, *Cq*DOP2, from *Culex quinquefasciatus*, the vector of lymphatic filariasis and West Nile virus.

**Methods/Results:**

*Cq*DOP2 has 94.7% amino acid identity to *Aa*DOP2 and 28.3% identity to the human D1-like DAR, hD1. *Cq*DOP2 and *Aa*DOP2 exhibited similar pharmacological responses to biogenic amines and DAR antagonists in cell-based assays. The antagonists amitriptyline, amperozide, asenapine, chlorpromazine and doxepin were between 35 to 227-fold more selective at inhibiting the response of *Cq*DOP2 and *Aa*DOP2 in comparison to hD1. Antagonists were toxic to both *C*. *quinquefasciatus* and *Ae*. *aegypti* larvae, with LC50 values ranging from 41 to 208 μM 72 h post-exposure. Orthologous DOP2 receptors identified from the African malaria mosquito, *Anopheles gambiae*, the sand fly, *Phlebotomus papatasi* and the tsetse fly, *Glossina morsitans*, had high sequence similarity to *Cq*DOP2 and *Aa*DOP2.

**Conclusions:**

DAR antagonists represent a putative new insecticide class with activity against *C*. *quinquefasciatus* and *Ae*. *aegypti*, the two most important mosquito vectors of NTDs. There has been limited change in the sequence and pharmacological properties of the DOP2 DARs of these species since divergence of the tribes Culicini and Aedini. We identified antagonists selective for mosquito versus human DARs and observed a correlation between DAR pharmacology and the *in vivo* larval toxicity of antagonists. These data demonstrate that sequence similarity can be predictive of target potential. On this basis, we propose expanded insecticide discovery around orthologous DOP2 targets from additional dipteran vectors.

## Author Summary

New mode-of-action insecticides are required to control arthropod vectors of neglected tropical diseases (NTDs). Rational drug design approaches offer attractive methods to identify new insecticidal chemistries that are potent and selective for molecular targets of arthropod vectors. Previously identified antagonists of a D_1_-like dopamine receptor (DAR) from the yellow fever mosquito, *Aedes aegypti* were toxic to the larvae of this species and are candidate novel insecticide leads. Building on this work, here we evaluated the molecular and pharmacological characteristics of an orthologous DAR from *Culex quinquefasciatus*, the vector of lymphatic filariasis and West Nile virus. We show that orthologous mosquito DARs have similar pharmacological profiles *in vitro* and that *Ae*. *aegypti*-active DAR antagonists are toxic to *C*. *quinquefasciatus* larvae *in vivo*. Sequence similarity between orthologous targets can be indicative of DAR target potential for discovery of potent, selective inhibitors. These findings justify expansion of insecticide discovery efforts to orthologous DARs from additional dipteran vectors of NTDs and provide support for DAR antagonists as a new class of chemistries for taxon-selective insecticides for vector control.

## Introduction

Arthropod vectors transmit six of the 17 neglected tropical diseases (NTDs) currently recognized by the World Health Organization (WHO). Of these, the causative agents of dengue virus and lymphatic filariasis are transmitted by mosquitoes in the subfamily Culicinae (Phylum Arthropoda; Class Insecta; Family Culicidae) and exact an enormous burden on human health in tropical and subtropical regions of the globe. *Aedes aegypti* is the principal vector of dengue, chikungunya, and yellow fever viruses, and *Culex quinquefasciatus* is the vector of West Nile virus and the nematode *Wuchereria bancrofti*, the causative agent of lymphatic filariasis. An estimated 50–100 million dengue infections occur annually [1] and approximately 120 million people are infected with *W*. *bancrofti* [2] with additional billions at risk of contracting these and other mosquito-borne diseases. Chikungunya is an ongoing threat in Africa and Southern Asia, and a recent outbreak could potentially lead to its establishment in the Americas [3].

The WHO has established a roadmap to eradicate multiple NTDs by 2020, backed by the London Declaration on Neglected Tropical Diseases [4, 5]. Achievement of this goal will require a multi-pronged, integrated approach involving new and existing vector control strategies, medicines, vaccines, and community outreach. Conventional insecticides will remain an important foundation of programs aimed at the control, elimination, and eradication of NTDs. Unfortunately the widespread development of insecticide resistant insect populations threatens continued control [6]. Vector control currently relies on a limited repertoire of active ingredients and the issue of insecticide cross-resistance is compounded by the fact that no new insecticides for insect vectors have become available for several decades [7]. In response, the Innovative Vector Control Consortium (IVCC) issued a call for three new insecticides with novel modes of action by 2023 to control malaria mosquitoes [8; http://www.ivcc.com]. The search for chemistries with unique and pest-specific modes of action with limited environmental impact necessitates new, rational design approaches [9].

**Fig 1:**
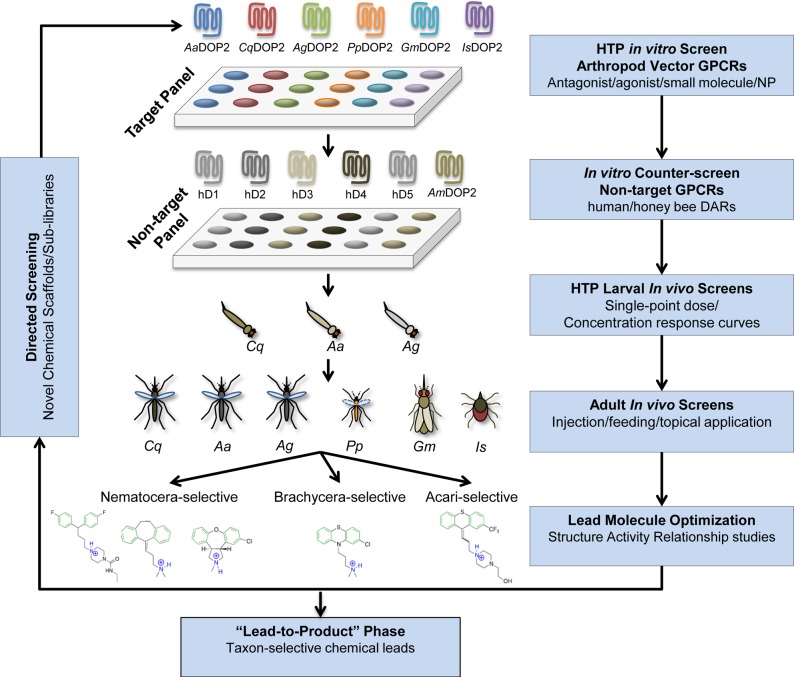
Schematic depicting PIDP activities aimed at discovery of D_1_-like DAR antagonists as new insecticides. The workflow is based on the evolving “genome-to-lead” component of the PIDP first described in Meyer et al. [11]. High-throughput (HTP), cell-based screens expressing arthropod D_1_-like DARs (Target Panel) are employed to identify chemistries active against one or more arthropod targets. Vector-selective chemistries are identified using counter screens expressing the human hD_1–5_ and the honeybee DAR (Non-target Panel). Subsequently, the *in vivo* toxicity of chemistries is confirmed in single-point dose and concentration response screens against mosquito larvae. Top hits are evaluated for activity against the adult stage of one or more vector species and taxon-level selectivity for the dipteran suborders Nematocera and Brachycera, and the subclass Acari. Information from structure activity relationship studies is used to direct iterative chemical screens. Chemical leads may enter the “Lead-to-Product” phase of the pipeline. New components of the pipeline described in the present study include the pharmacologically characterized *Cq*DOP2 target, the *Ag*DOP2, *Pp*DOP2 and *Gm*DOP2 targets identified from assembled genome sequences (S2 Fig), and the *C*. *quinquefasciatus* larval screen. Remaining components are the subject of works in review [15] and ongoing efforts. Abbreviations: *Aa*, *Aedes aegypti; Ag*, *Anopheles gambiae; Am*, *Apis mellifera*; *Cq*, *Culex quinquefasciatus; Gm*, *Glossina morsitans; Is*, *Ixodes scapularis; Pp*, *Phlebotomus papatasi;* NP, natural product.

G protein-coupled receptors (GPCRs) are successful pharmaceutical targets with over one third of human drugs acting on these receptors or their downstream signaling processes [10]. Invertebrate GPCRs have long been suggested as targets for the development of new classes of insecticides [11, 12]. The Purdue Insecticide Discovery Pipeline (PIDP) [13] is a GPCR-based platform established for discovery and development of novel mode-of-action insecticides for vector control [11, 13, 14, 15]. Initially the PIDP is pursuing small molecule antagonists and agonists of invertebrate dopamine receptors (DARs) (Fig. 1) and has demonstrated proof of concept in the *Ae*. *aegypti* DAR system [11, 13]. Vertebrate and invertebrate DARs are biogenic amine receptors in the Class A rhodopsin-like subfamily of GPCRs. DARs have been implicated in several neurological diseases of humans such as Parkinson's disease and schizophrenia. Scientific investment in human DAR pharmacology and associated therapeutic interventions [16, 17] provides a much needed foundation to drive equivalent discovery work in arthropod systems.

Dopamine has a role in locomotion, learning, courtship, development, and several other complex behaviors in invertebrates [see 18, 19, and 11 for an overview]. Several studies suggest that interference with dopaminergic processes may cause insect death or result in a variety of phenotypes such as incapacitation and disrupted development [11, 13, 15, 20, 21] that are highly attractive for insecticide development. The rational design of invertebrate DAR- and other GPCR-targeting chemistries could generate highly effective molecules for vector control. Invertebrates typically possess two D_1_-like receptors (Gα_s_ coupled) and a single D_2_-like receptor (Gα_i/o_ coupled) [19, 22]. One of the D_1_-like DARs, hereafter referred to as DOP2, exhibits high amino acid sequence identity among the arthropods *Ae*. *aegypti*, *Anopheles gambiae* (African malaria mosquito), *C*. *quinquefasciatus* (northern house mosquito), *Ixodes scapularis* (Lyme disease tick), *Drosophila melanogaster* (fruit fly), and *Apis mellifera* (honey bee) across the transmembrane (TM) spanning domains (>70%) but limited sequence identity to the human D_1_-like DARs, hD_1_ and hD_5_ (<55%) [13]. Two D_1_-like DAR sequences, *Aa*DOP1 and *Aa*DOP2, were identified in the genome of the yellow fever mosquito, *Ae*. *aegypti* [23]. Assays using *Aa*DOP1 or *Aa*DOP2 expressing cells revealed elevated cAMP levels following exposure to dopamine in a concentration-dependent manner, providing support for the classification of these receptors [11]. Subsequently, the discovery of receptor antagonists with mosquitocidal properties was demonstrated by screening chemical libraries for *Aa*DOP2 antagonists in cell-based assays [11, 13]. Antagonists were evaluated in hit-to-lead studies that showed multiple compounds were selective for the *Aa*DOP2 target versus hD_1_ and caused rapid and high mortality of *Ae*. *aegypti* larvae *in vivo*.

Two D_1_-like DARs, *Cq*DOP1 and *Cq*DOP2 were identified from the assembled genome of *C*. *quinquefasciatus* [24] with *Cq*DOP2 identified as the ortholog to *Aa*DOP2 on the basis of amino acid similarity [13]. *Aa*DOP2 and *Cq*DOP2 provide an opportunity to address questions of relevance to insecticide discovery, namely (1) does sequence similarity between orthologous targets equate to conservation of pharmacological properties *in vitro*, (2) is sequence similarity predictive of the toxicity of target inhibitors *in vivo*, and (3) can differences in sequence between targets be exploited for development of taxon-selective chemistries? Here we present the first study to assess conservation in the molecular and pharmacological properties of orthologous dopamine receptors from species representing two of the most important mosquito genera affecting human health, *Culex* and *Aedes*. The human receptor, hD_1_, was evaluated in parallel to determine the relative potency and selectivity of DAR antagonists for mosquitoes versus humans. *Aa*DOP2 antagonists were evaluated for toxicity to larvae of *C*. *quinquefasciatus* to explore the potential of extending the DAR antagonist-based insecticide discovery approach to this vector. Lastly, *in silico* analyses were conducted to evaluate DOP2 targets from three additional dipteran vectors of NTDs for inclusion in the PIDP; the *An*. *gambiae* mosquito, a vector of malaria, the *Phlebotomus papatasi* sand fly vector of leishmaniasis, and the *Glossina morsitans* tsetse fly vector of Human African Trypanosomiasis (sleeping sickness).

## Methods

### Mosquito culture

Mosquito larvae of the Liverpool strain IB12 of *Ae*. *aegypti* and the Johannesburg strain of *C*. *quinquefasciatus* were reared in an insectary on a 12 h day/night cycle at 75% RH at 28°C in 25 x 40 cm plastic pans (150 larvae per pan) on hamster pellets (*Ae*. *aegypti*) or ground flake fish food (*C*. *quinquefasciatus*). Adult *Ae*. *aegypti* and *C*. *quinquefasciatus* that eclosed under this rearing regimen had an average wing length of 3.4 mm (measured as in [25]) and 3.2 mm (measured as in [26]), respectively, suggesting diet was sufficient and larval crowding effects were minimal.

### *Cq*DOP2 receptor sequence and molecular characterization

The amino acid sequences of DARs from multiple arthropods were used to search the *C*. *quinquefasciatus* genome using the Basic Local Alignment Search Tool (tBLASTn, [27]). Gene models were confirmed by sequencing of amplified RT-PCR products following procedures described in Meyer et al [11]. Briefly, total RNA was extracted from adult *C*. *quinquefasciatus* females and treated with RNase-free DNase. RT-PCR amplification was performed using the SuperScript One-Step RT-PCR kit (Invitrogen, Carlsbad, CA) and the *Cq*DOP2-specific primers CqDOP2_1F: 5'-ATGATGACTACGAATGCAACTGATTAC-3' and CqDOP2_1R: 5'-CTAAATGTACGTCTGCTCGCAC-3'. RT-PCR products separated by electrophoresis on a 1% agarose gel were excised and cloned using the TOPO TA cloning kit (Invitrogen, Carlsbad, CA). Purified plasmids from the resulting clones were sequenced at the Purdue Genomics Core Facility (PGFC). DNA sequences were used to predict full-length coding regions and manual annotation was performed using Artemis software (version 9) [28]. The *Cq*DOP2 conceptual protein sequence was aligned to that of *Aa*DOP2 using ClustalW [29] and used to identify conserved amino acid residues and predict protein structural features [22, 30, 31].

To determine receptor expression in different life stages, total RNA was isolated from *C*. *quinquefasciatus* eggs, L4 larvae, pupae, and 5-day old adults (female and male) using the RNeasy Mini Kit (Qiagen) and following kit protocols, including DNase treatment. Generation of cDNA was performed using the iScript cDNA Synthesis Kit (Bio-Rad). Reverse transcription polymerase chain reaction (RT-PCR) was used with cDNA template, primers CqDOP2_1F and CqDOP2.2R: 5'-CCAGCAGTGGAAGATAGAACG-3', Taq polymerase (Phusion, New England Biolabs) and the following thermo-cycling conditions: 35 cycles at 94°C, 45 s; 55°C, 45 s; 72°C, 2 min. Subsequent products were separated by gel electrophoresis and photographed (EpiChemi II Darkroom, UVP Laboratory Products). Products of approximately 700 bp in length were excised, purified (MinElute Gel Extraction Kit, Qiagen), and sequenced at the PGCF.

A neighbor-joining phylogenetic analysis was conducted using amino acid sequences of arthropod and mammalian GPCRs retrieved from GenBank. MEGA6 [32] was used to align and perform tree construction according to the procedure of Hall [33]. Diuretic hormone 44 receptor 1 (*D*. *melanogaster*) was used as an outgroup. Bootstrap analysis (1000 replicates) was performed as an estimate of branch reliability.

### *In silico* assessment of DOP2 DAR targets from additional dipteran vectors of NTDs

To assess the potential of expanding the PIDP pipeline to orthologous DOP2 targets from a range of key dipteran vectors, additional tBLASTn searches of the assembled genomes of *An*. *gambiae*, *P*. *papatasi* (www.vectorbase.org) and *G*. *morsitans* [34] were performed using *Aa*DOP2 and *Cq*DOP2 sequences. The conceptual amino acid sequences for the resultant gene models, *Ag*DOP2, *Pp*DOP2, and *Gm*DOP2, were aligned with *Aa*DOP2 and *Cq*DOP2 using ClustalW [29] and conserved structural features were predicted as described above.

### Pharmacological characterization of *Cq*DOP2 and *Aa*DOP2

For functional characterization of the mosquito receptors, *Cq*DOP2 and *Aa*DOP2 were synthesized by Genscript (Piscataway, NJ) and cloned into the expression vector pcDNA3.1+ (Invitrogen, Carlsbad, CA). Stable cell lines expressing the receptors in HEK293 cells were generated as described previously for *Aa*DOP2 [11]. The *Aa*DOP2 expressing cells used here were from the same clone previously utilized for *Aa*DOP2 characterization [11]. Briefly, HEK293-CRELuc cells were plated in Dulbecco's modified eagle's medium (DMEM) supplemented with 5% bovine calf serum, 5% fetal clone I (Thermo Scientific, Waltham MA), 1% Antibiotic-Antimycotic (Life Technologies, Grand Island NY) and 2 μg/ml puromycin (Sigma-Aldrich, St. Louis, MO), transfected, and then subjected to selection with G418 (600 μg/ml). G418-resistant clones were selected and screened for receptor expression in the cAMP activated luciferase (CRELuc) reporter cell line construct [11]. For pharmacological characterization of receptor activity, cryopreserved cells stably expressing *Aa*DOP2 (10,000 cells/well), *Cq*DOP2 (5,000 cells/well), or human D_1_ (5,000 cells/well) were thawed, washed, and re-suspended in assay buffer (Hank’s balanced salt solution, Hyclone, Logan, UT) supplemented with 20 mM 2-[4-(2-hydroxyethyl)piperazin-1-yl]ethanesulfonic acid (HEPES, Hyclone, Logan, UT) and 0.1% bovine serum albumin (MP Biomedicals, Santa Ana, CA), seeded in white 384 well plates (PerkinElmer, Waltham, MA), and incubated for 1 h at 37°C. Compounds were serially diluted in assay buffer containing 3-isobutyl-1-methylxanthine (IBMX, final concentration 0.5 mM), added to the plates, and incubated for 1 h at 25°C to allow for cAMP accumulation. Reactions were stopped and cAMP was measured by a homologous time-resolved fluorescence (HTRF) assay according to the manufacturer’s recommendations (Cisbio, Bedford, MA). Fluorescence was read on a Synergy4 plate reader (BioTek, Winooski, VT).

Dopamine hydrochloride, histamine dihydrochloride, 5-hydroxytryptamine hydrochloride (serotonin), (±)-octopamine hydrochloride, tyramine hydrochloride (Sigma-Aldrich, St. Louis, MO), (-)-epinephrine bitartrate, and L (-)-norepinephrine bitartrate (Research Biochemical International, Natick, MA) were used for initial receptor characterization studies. Antagonist profiles were generated by adding serially diluted antagonists followed by dopamine (3 μM for *Aa*DOP2- and *Cq*DOP2-, and 0.5 μM for hD_1_-expressing cells). The antagonists amitriptyline hydrochloride, asenapine maleate, (±) butaclamol hydrochloride, chlorpromazine hydrochloride, doxepin hydrochloride, cis-(Z)-flupenthixol dihydrochloride, SCH23390 hydrochloride (Sigma-Aldrich, St. Louis, MO), and amperozide hydrochloride (Tocris bioscience, Ellisville, MO) were selected based on previous chemical screens against *Aa*DOP2 and subsequent bioassays against *Ae*. *aegypti* larvae [11, 13]. All serial dilutions were carried out using the Precision liquid handling station (BioTek, Winooski, VT). Data were collected from a minimum of three independent experiments conducted in duplicate. Statistical analysis of data was conducted with GraphPad Prism 6 software (GraphPad Software Inc., San Diego, CA).

### *In vivo C*. *quinquefasciatus* and *Ae*. *aegypti* bioassays

A panel of nine *Aa*DOP2 antagonists was selected based on toxicity determined from single-point dose, high-throughput screens against *Ae*. *aegypti* larvae [13, 15]. Compounds were evaluated in parallel in concentration-response assays against third-instar larvae (L3) of *C*. *quinquefasciatus* and *Ae*. *aegypti* at room temperature (23–25°C). Larvae were transferred using a plastic pipette to the wells of a 24-well tissue culture plate (Corning Inc., Corning NY) (five larvae per well) containing 1 ml de-ionized water and 400, 200, 100, 50, or 25 μM test compound or water only as the control. Antagonists were diluted in water to the desired concentration immediately before transfer to tissue culture plates. Larval mortality was determined every 30 min for the first 3 h, then daily at 24, 48, and 72 h post-exposure. Plates were gently shaken and larvae were lightly touched with a sterile probe (up to three times, as required) and stringent criteria were established for scoring such that larvae that failed to respond to both stimuli were recorded as dead. Four technical replicates were performed per dose, and each bioassay was performed a minimum of three times. Calculations of lethal concentration 50 (LC_50_) and lethal time 50 (LT_50_) were made using GraphPad Prism 6 software (GraphPad Software Inc., San Diego, CA).

## Results

### *Cq*DOP2 receptor sequence and molecular characterization

**Fig 2:**
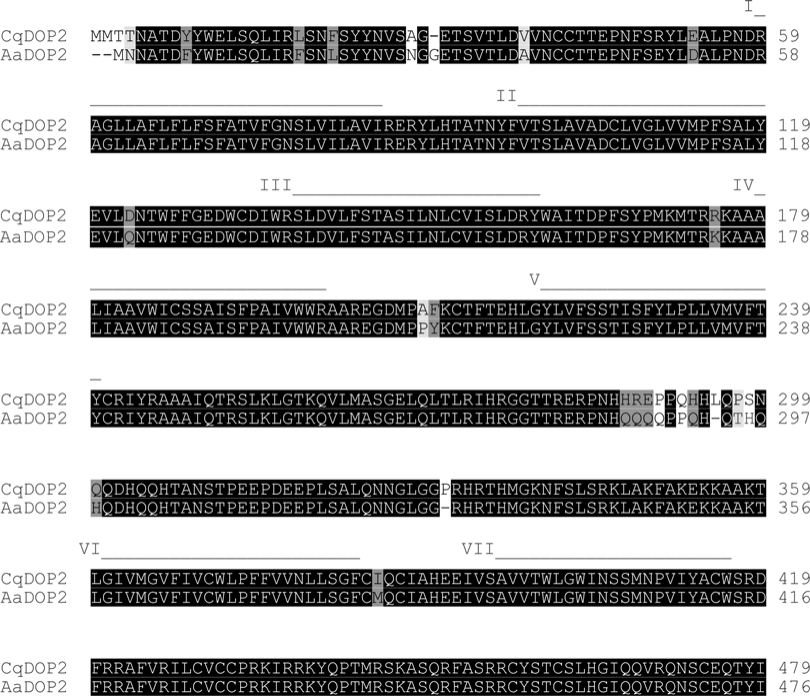
Alignment of *Cq*DOP2 and *Aa*DOP2 amino acid sequences. Highlighted areas indicate identical and conserved residues as designated by ClustalW [29]: black = identical residues; dark gray = strongly similar residues; light gray = weakly similar residues (for amino acid similarity groups, see: http://www.clustal.org/download/clustalx_help.html). Putative transmembrane (TM) domains I-VII are indicated as a line above the alignment.

A 1,440 bp sequence encoding the predicted open reading frame of *Cq*DOP2 was identified from cloned RT-PCR products (Genbank ID KM262648). Alignment of the conceptual *Cq*DOP2 amino acid sequence with that of *Aa*DOP2 showed high overall amino acid sequence identity (94.7%) and 100% identity in the predicted TM-spanning domains (Fig. 2). The greatest divergence between the two sequences was observed in the third intracellular loop (ILIII) with nine amino acid differences and two additional amino acids in *Cq*DOP2, and in the N-terminus with seven differences, and two additional amino acids in *Cq*DOP2 and one additional residue in *Aa*DOP2. The remaining amino acid differences were primarily conservative substitutions. *Cq*DOP2 possesses key biochemical features considered essential for GPCR function and that were also identified in *Aa*DOP2 (Table 1). Residues D140, S225, and S228 of *Cq*DOP2 are predicted to interact directly with biogenic amines [19] while the "DRY" motif (residues 157–159), and aspartate in TMII (D105) are required for receptor activation. *Cq*DOP2 also possesses several putative palmitoylation and phosphorylation sites. Sequences from ~700 bp RT-PCR products amplified from *C*. *quinquefasciatus* eggs, L4 larvae, pupae or adult male or female cDNA (S1 Fig) matched that of the expected region of *Cq*DOP2, confirming the presence of *Cq*DOP2 transcripts in the life stages examined.

**Table 1:** Comparison of protein features for *Cq*DOP2 and *Aa*DOP2.

Protein features	*Cq*DOP2	*Aa*DOP2^a^
Total length	479	476
Length of N-terminus^b^	58	57
Length of intracellular loops I, II, III^b^	10, 20, 117	10, 20, 115
Length of extracellular loops I, II, III^b^	15, 18, 9	15, 18, 9
Length of carboxyl tail^b^	63	63
1–4 N-linked glycosylation sites (N-terminus)	N5, N21, N26, N47	N3, N19, N24, N46
Conserved cysteines in extracellular loops 1–2^c^	C133, C212	C132, C211
C-terminus palmitoylation sites (C)	C387, C431, C432, C457	C384, C428, C429, C454
Protein kinase A/C phosphorylation (Intracellular loops II, III and C-terminus)	S167, T173, T251, S253, T279, T307, S342, S344, T443, S449, S454, S459, T460, S462, S473	S166, T172, T250, S252, T278, T305, S339, S341, T440, S446, S451, S456, T457, S459, S470
Conserved aspartates in TMII, TMIII^d^	D105, D140	D104, D139
Conserved “DRY” motif^e^	D157, R158, Y159	D156, R157, Y158
Conserved serines in TMV^f^	S224, S225, S228	S223, S224, S227
Conserved aromatic residue in TMV^g^	F229	F228
Conserved aromatic residues in TMVI^g^	W371, F374, F375	W368, F371, F372

The number of amino acids composing the N- and C-termini and the intracellular and extracellular loops are relative to the transmembrane domain (TM) sequences shown in Fig. 2.

^a^ *Aa*DOP2 features from [11]

^b^Values refer to the number of amino acids composing these features

^c^Presumed to form a disulfide bond for protein stabilization

^d^Predicted as important for binding the amine moieties of catecholamines

^e^Implicated in G-protein coupling

^f^Predicted to form hydrogen bonds with catechol hydroxyl groups

^g^Aromatic residues implicated in ligand interaction

**Fig 3:**
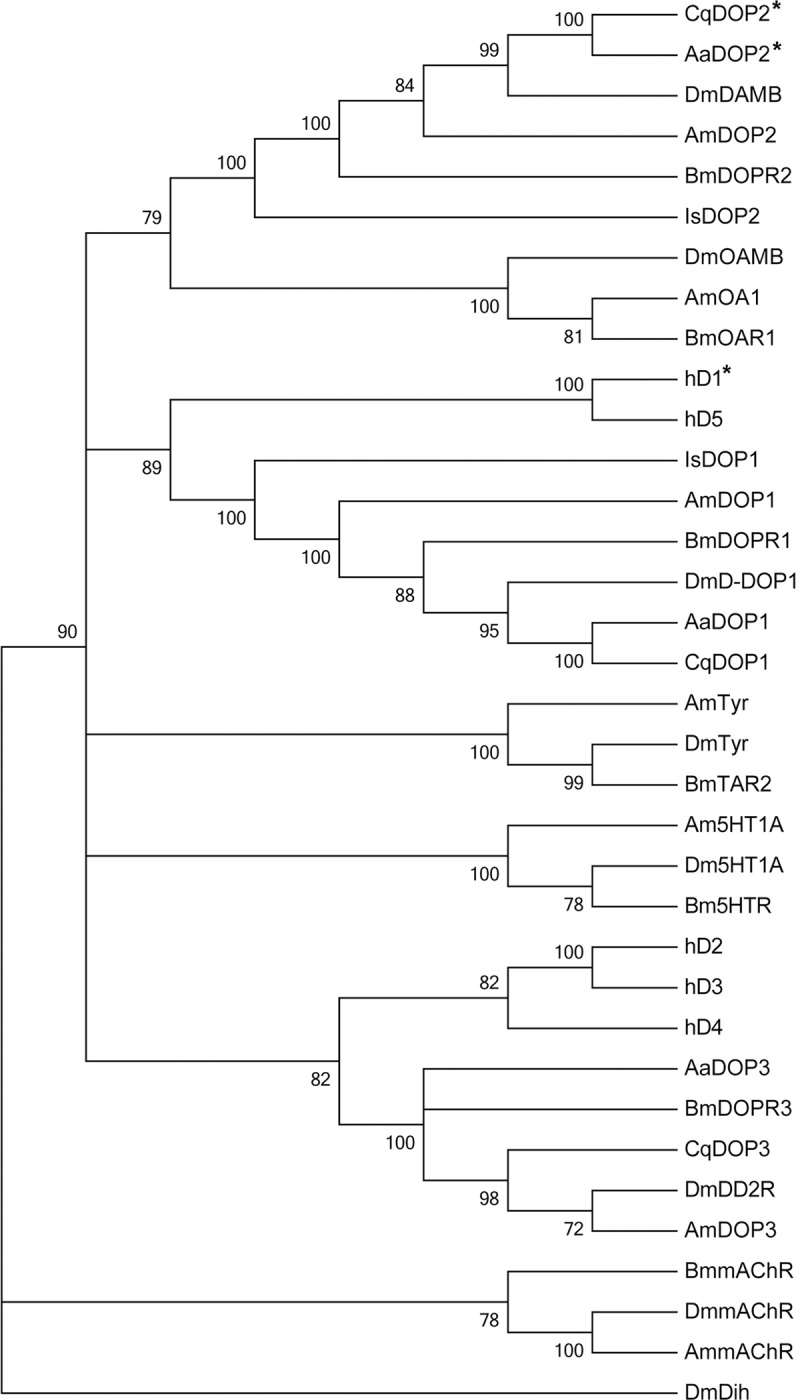
Neighbor-joining sequence analysis of *Cq*DOP2, *Aa*DOP2 and representative biogenic amine receptors. Abbreviations and NCBI accession numbers of species indicated are as follows: *Aedes aegypti* = Aa; AaDOP1 = D_1_-like dopamine receptor 1 (JN043502); AaDOP2 = D_1_-like dopamine receptor 2 (JN043503); AaDOP3 = D_2_-like dopamine receptor (XM_001648573); *Culex quinquefasciatus* = Cq; CqDOP1 = D_1_-like dopamine receptor 1 (XM_001842358); CqDOP2 = D_1_-like dopamine receptor 2 (KM262648); CqDOP3 = D_2_-like dopamine receptor (XM_001865540); *Ixodes scapularis* = Is; IsDOP1 = D_1_-like dopamine receptor 1 (ISCW001496); IsDOP2 = D_1_-like dopamine receptor 2 (ISCW008775); *D*. *melanogaster* = Dm; DmD-Dop1 = D_1_-like dopamine receptor (P41596); DmDAMB = D_1_-like dopamine receptor (DopR99B/DAMB: AAC47161); DmDD2R = D_2_-like dopamine receptor (DD2R-606: AAN15955); DmDih = diuretic hormone 44 receptor 1 (NP_610960.1); DmmAChR = muscarinic acetylcholine receptor (AAA28676); DmOAMB = octopamine receptor in mushroom bodies, isoform A (NP_732541); Dm5HT1A = serotonin receptor 1A, isoform A (AAM68432); DmTyr = tyramine receptor (CG7431: NP_650652); *Apis mellifera* = Am; AmDOP1 = D_1_-like dopamine receptor (NP_001011595); AmDOP2 = D_1_-like dopamine receptor (NP_001011567); AmDOP3 = D_2_-like dopamine receptor (NP_001014983); AmmAChR = muscarinic acetylcholine receptor (XP_395760); AmOA1 = octopamine receptor (oar, NP_001011565); Am5HT1A = serotonin receptor (NP_001164579); AmTyr = tyramine receptor (NP_001032395.1); *Bombyx mori* = Bm; BmDOPR1 = D_1_-like dopamine receptor (AB162715); BmDOPR2 = D_1_-like dopamine receptor (AB162716); BmDOP3 = D_2_-like dopamine receptor (XM_004925908); BmmAChR = muscarinic acetylcholine receptor (XM_004922849); BmOAR1 = octopamine receptor (NM_001098278); Bm5HTR = serotonin receptor (X95604); BmTAR2 = tyramine receptor (NM_001171178); *Homo sapiens* = h; hD1, D_1_-like dopamine receptor (D(1A), NP_000785); hD2 = D_2_-like dopamine receptor (D(2), NP_000786); hD3 = D_2_-like dopamine receptor (D(3), NP_000787); hD4 = D_2_-like dopamine receptor (D(4), NP_000788); hD5 = D_1_-like dopamine receptor (D(1B)/D5,NP_000789). * Indicates receptors pharmacologically characterized in the current study.

Neighbor-joining sequence analysis (Fig. 3) placed *Cq*DOP2 in a clade with other invertebrate D_1_-like DOP2 receptors from *Ae*. *aegypti* (*Aa*DOP2), *Ap*. *mellifera* (*Am*DOP2), *B*. *mori* (*Bm*DOPR2), *D*. *melanogaster* (*Dm*DAMB), and *I*. *scapularis* (*Is*DOP2). This group formed part of a larger clade comprising invertebrate octopamine receptors (*Dm*OAMB, *Bm*OAR1, and *Am*OA1), but not human DARs. The analysis also revealed a second cluster comprising other invertebrate D_1_-like receptors, including *Is*DOP1, *Am*DOP1, *Bm*DOPR1, *Dm*D-DOP1, and *Aa*DOP1 and the human DARs, hD_1_ and hD_5_. The invertebrate D_2_-like receptor sequences from *Ap*. *mellifera* (*Am*DOP3), *B*. *mori* (*Bm*DOPR3), and *D*. *melanogaster* (*Dm*DD2R) were placed in a cluster with *Cq*DOP3 and *Aa*DOP3 and formed part of a larger clade with the human D_2_-like receptors hD_2_, hD_3_, and hD_4_.

### *In silico* assessment of DOP2 DAR targets from additional dipteran vectors of NTDs

Sequences containing putative DOP2 coding regions were identified from *An*. *gambiae* (*Ag*DOP2), *P*. *papatasi* (*Pp*DOP2), and *G*. *morsitans* (*Gm*DOP2) using tBLASTn searches. Percentage identity of amino acid sequences relative to *Aa*DOP2 were as follows: *Ag*DOP2 = 82.6%; *Pp*DOP2 = 81.3%; *Gm*DOP2 = 79.0%. Alignment revealed preservation of aspartate and serine residues predicted to bind biogenic amines and key aspartate residues and the DRY motif required for receptor activation (S2 Fig). Putative palmitoylation and phosphorylation sites were also preserved with the exception of residue K426 in *P*. *papatasi*. Amino acid sequences were most divergent between species in the N-terminus and in ILIII.

### Pharmacological characterization of *Cq*DOP2 and *Aa*DOP2

**Fig 4:**
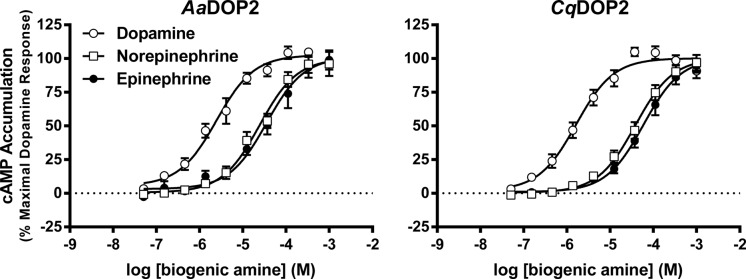
Pharmacological characterization of *Aa*DOP2 and *Cq*DOP2 stably expressed in HEK293 cells. Normalized cAMP response (mean ± SEM) seen as a function of concentration of dopamine, norepinephrine, and epinephrine for each receptor. The graphs are based on the compiled data (n ≥ 8 independent experiments, conducted in duplicate) and normalized using GraphPad Prism software to the maximal dopamine response for each experiment.

Although we have previously reported partial characterization of *Aa*DOP2 using a luciferase-based system [11], we evaluated *Cq*DOP2 and *Aa*DOP2 here in parallel using a HTRF-based cAMP assay (Cisbio, Bedford, MA) to avoid assay-induced bias. Both *Cq*DOP2 and *Aa*DOP2 responded to dopamine, with EC_50_ values of 2.3 and 1.7 μM, respectively (Fig. 4). For both receptors, epinephrine and norepinephrine elicited an increase in cAMP, however, these biogenic amines were much less potent having EC_50_ values at least 10-fold higher than that of dopamine (Table 2). Treatment with histamine, octopamine, serotonin, or tyramine did not cause a measurable response in activity for either receptor.

**Table 2:** EC_50_ values (μM±SEM) for dopamine and other biogenic amines estimated from concentration response curves measuring the cAMP response from HEK293 cells expressing *Aa*DOP2 or *Cq*DOP2 (n ≥ 8 conducted in duplicate).

	*Aa*DOP2	*Cq*DOP2
Dopamine	3.4±0.9	2.1±0.4
Epinephrine	41.1±10.5	80.8±21.0
Norepinephrine	30.3±7.5	52.2±13.8

No response was observed for histamine, octopamine, serotonin, or tyramine for concentrations up to 100 μM.

**Fig 5:**
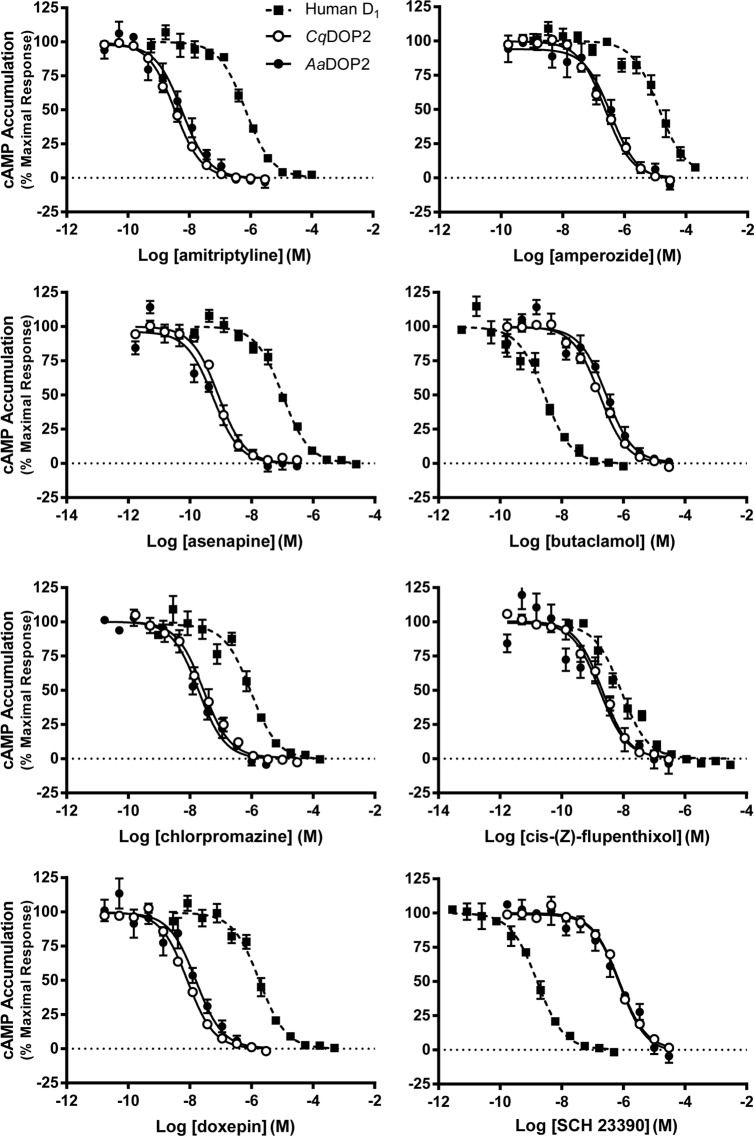
Characterization of select antagonists on receptor activity in HEK cells stably expressing *Cq*DOP (○), *Aa*DOP2 (●), or the human D_1_ receptor (▪). Graphs are based on compiled cAMP measurements (n ≥ 3 independent experiments) normalized using GraphPad Prism software to the dopamine response for each experiment and shown as mean ± SEM.

The dopamine-stimulated activity of *Cq*DOP2 and *Aa*DOP2 in response to a subset of *Aa*DOP2 and hD_1_ antagonists mirrored each other, but differed from those observed for hD_1_ (Fig. 5, Table 3). Amitriptyline, asenapine, amperozide, chlorpromazine, and doxepin were markedly more potent at both mosquito receptors over the human receptor. In contrast, the D_1_ antagonists (±) butaclamol and SCH23390 were approximately 60-fold and 500-fold more selective for the human receptor over the mosquito receptors, respectively.

**Table 3:** IC_50_ values (nM±SEM) for inhibition of dopamine-stimulated cAMP response in HEK293 cell lines by DAR antagonists.

	*Aa*DOP2	*Cq*DOP2	Human D_1_	Fold selectivity to Human D_1_	
				*Aa*DOP2	*Cq*DOP2
Amitriptyline	6.4±2	3.3±0.4	690±52	108	209
Amperozide	469±71	248±42	16200±4770	35	65
Asenapine	0.7±0.2	0.8±0.03	101±2	144	126
(±)-Butaclamol	226±36	172±15	2.8±0.5	0.012	0.016
Chlorpromazine	19±1	7.4±2	972±160	51	131
Cis-(Z)-flupenthixol	2±0.8	2±0.5	9.4±3	5	5
Doxepin	17±4	8.3±0.6	1890±455	111	227
SCH23390	709±187	745±62	1.6±0.2	0.002	0.002

Values were determined from concentration response curves measuring the cAMP response. Fold selectivity was determined by dividing human D_1_ IC_50_ values by each respective mosquito DOP2 IC_50_ value for each antagonist.

### *In vivo C*. *quinquefasciatus* and *Ae*. *aegypti* bioassays

**Fig 6:**
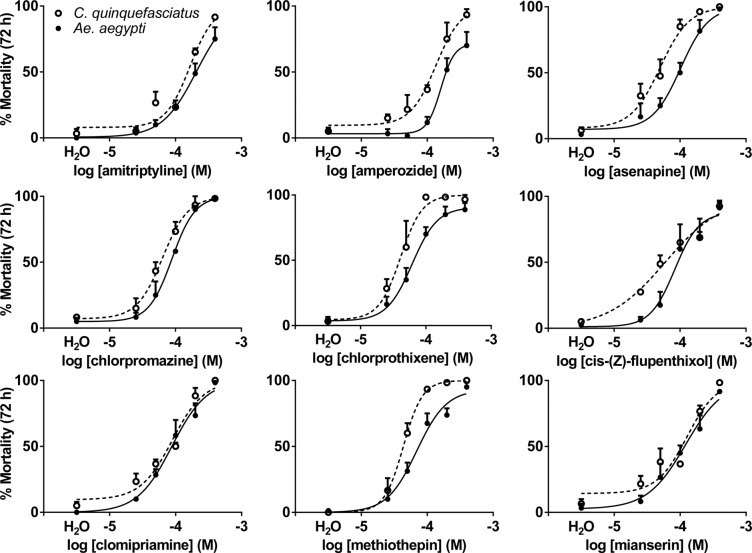
Concentration response curves for *C*. *quinquefasciatus* (○) and *Ae*. *aegypti* (●) showing percent larval mortality at 72 h post exposure to DOP2 antagonists. Each data point represents mean ± SEM (n ≥ 3 independent experiments).

To test the *in vivo* activity of select antagonists, concentration-response assays were conducted against L3 larvae of *C*. *quinquefasciatus* and *Ae*. *aegypti*. All DAR antagonists elicited ≥70% mortality of *Ae*. *aegypti* and *C*. *quinquefasciatus* larvae by 72 h at 400 μM, the highest dose tested (Fig. 6). The LC_50_ values at 72 h ranged from 41 to 208 μM depending on antagonist and mosquito species (Table 4). Chlorprothixene was the most toxic compound, eliciting the lowest LC_50_ values in both species (41±7 μM for *C*. *quinquefasciatus* and 62±9 μM for *Ae*. *aegypti*) and the lowest LT_50_ values (13.9±2.0 h for *C*. *quinquefasciatus* and 22.2±3.2 h for *Ae*. *aegypti*) (Table 4). Mortality to antagonists in both species was similar, although *C*. *quinquefasciatus* larvae were slightly more susceptible than *Ae*. *aegypti* larvae, having lower LT_50_ and LC_50_ values (Fig. 6, Table 4). Four compounds (chlorprothixene, chlorpromazine, methiothepin, and mianserin) caused >70% mortality in *C*. *quinquefasciatus* within the first 24 h (S3 Fig), and all but amitriptyline caused >70% mortality by 48 h (S4 Fig). In *Ae*. *aegypti*, >70% mortality was observed after 48 h for five compounds (chlorprothixene, chlorpromazine, methiothepin, mianserin, and asenapine) (S4 Fig).

**Table 4:** Lethal concentration (LC_50_) values (μM±SEM, 72 h exposure) and lethal time (LT_50_) values (h±SEM at 400 μM), for *Aa*DOP2 antagonists on larvae of *C*. *quinquefasciatus* (*Cq*) and *Ae*. *aegypti* (*Aa*) (n ≥ 3).

	LC_50_		LT_50_	
	***Cq***	***Aa***	***Cq***	***Aa***
Amitriptyline	165±37	208±137	41.7±2.1	47.8±6.3
Amperozide	135±35	161±20	25.4±1.5	46.6±6.2
Asenapine	50±10	102±22	24.2±1.3	36.4±1.6
Chlorpromazine	64±9	88±9	16.7±1.1	26.1±0.9
Chlorprothixene	41±7	62±9	13.9±2.0	22.2±3.2
Cis-(Z)-flupenthixol	59±22	83±25	35.2±6.1	42.6±2.9
Clomipramine	89±19	88±24	27.3±3.0	28.7±3.1
Methiothepin	44±3	69±12	15.7±4.9	30.6±1.4
Mianserin	126±46	121±57	17.9±3.0	22.8±1.3

## Discussion

The sequence identity (94.7%, Fig. 2), key structural features (Table 1), and pharmacological profile (Figs. 4–5, Tables 2–3) of *Cq*DOP2 identify this receptor as the ortholog of *Aa*DOP2 (Figs. 2, 3). The detection of *Cq*DOP2 (S1 Fig) and *Aa*DOP2 transcripts [11] in all life stages examined suggests these receptors are constitutively expressed and likely regulate essential, conserved functions throughout the insects' life cycle. The 24 amino acid substitutions (the majority of which are conservative), additions, or deletions between the two sequences (Fig. 2) provided an opportunity to assess impact of these residues on DAR pharmacology. The similar pharmacological profiles of *Cq*DOP2 and *Aa*DOP2 suggest that the 24 residues in question do not greatly affect receptor interaction with either dopamine or a variety of antagonists. DOP2 was likely present in the common ancestor of modern mosquitoes and ticks as all invertebrate D_1_-like DOP2 sequences clustered together in the neighbor-joining analysis (Fig. 3). The overall amino acid identity of DOP2 DARs from *An*. *gambiae*, *P*. *papatasi* and *G*. *morsitans* relative to *Aa*DOP2 decreased as taxonomic distance increased between the subfamily Culicinae and the subfamily Anophelinae (*Ag*DOP2, 82.6%), and the dipteran suborders Nematocera (*Pp*DOP2, 81.3%) and Cyclorrhapha (*Gm*DOP2, 79.0%). The pharmacological characterization of these receptors (S2 Fig), together with published data for *Is*DOP2 from the tick, *I*. *scapularis* (Fig. 1) [14, 22], will contribute to an understanding of how progressively greater changes in amino acid sequence influence pharmacology between DARs from arthropod vectors of divergent lineages.

*Cq*DOP2 was pharmacologically distinguished as a D_1_-like dopamine receptor through heterologous expression experiments that showed concentration-dependent increases in cAMP production in response to dopamine. Responses were also elicited by epinephrine and norepinephirine but over 10-fold more agonist was required to reach maximum stimulation of cAMP in comparison to dopamine (Fig. 4, Table 2). *Cq*DOP2 displayed a similar pharmacological profile to *Aa*DOP2 in the presence of dopamine, epinephrine, and norepinephirine, suggesting that the dopaminergic ligand binding pocket and receptor activation have been conserved between these species. In *Ap*. *mellifera*, a response to norepinephirine was also noted for *Am*DOP2, requiring approximately 25-fold more agonist than dopamine [19]. The *D*. *melanogaster* DOP2 ortholog, *Dm*DAMB, also was modestly receptive to norepinephirine. Neither of the mosquito receptors in the current study showed detectable cAMP production over basal in response to histamine, serotonin, tyramine, or octopamine, a result similar to characterizations of *Ap*. *mellifera* and *D*. *melanogaster* DOP2 receptors [19]. Slight discrepancies between dopamine EC_50_ values for *Aa*DOP2 in this study (2.3 ± 0.7 μM) and previous reports (0.24 ± 0.16 μM) may reflect the different assay methods used; HTRF in the current study and a luciferase-based reporter assay in Meyer et al. [11]. The HTRF assay directly measures cAMP and offers enhanced accuracy over the indirect cAMP measurements of the luciferase assay [15]. In addition, adoption of the HTRF assay into the PIDP (Fig. 1) is seen as a technical improvement for high throughput screening.

The response of *Cq*DOP2 and *Aa*DOP2 to antagonists was also similar (Fig. 5, Table 3). In contrast, hD_1_ was much less sensitive to amitriptyline, asenapine, amperozide, chlorpromazine, and doxepin and more sensitive to (±) butaclamol and SCH23390 than either mosquito receptor (Table 3). This pharmacological evidence is in agreement with previous studies that show a structural and pharmacological distinction between the five human DARs and arthropod DOP2 receptors [19, 35]. The difference in activity between the mosquito and human receptors in our study suggests antagonists could be developed with target activity for insect pests while remaining safe for non-target organisms, although further testing is required. The response to cis-(Z)-flupenthixol was similar across all three receptors (Fig. 5, Table 3) suggesting overlapping antagonist interactions between these divergent DARs. Cis-(Z)-flupenthixol has previously been noted as a nonselective DAR antagonist in arthropods [19] highlighting the importance of counter screening to avoid pursuit of leads with potential effects on non-target organisms (See Fig. 1).

Consistent with the findings of Meyer et al. [11] and Hill et al. [13], all DAR antagonists were highly toxic to *Ae*. *aegypti* larvae *in vivo*, and demonstrated similar toxicity profiles against *C*. *quinquefasciatus* larvae. This is the first report of DAR antagonist toxicity to the latter species. Larval assays revealed a similar rank order of DAR antagonist-induced mortality to that observed for the potency of antagonists at *Cq*DOP2 and *Aa*DOP2 *in vitro*, with the exception of amitriptyline in both species and asenapine in *Ae*. *aegypti*. These results suggest that small molecule DOP2 antagonist leads identified for either *Ae*. *aegypti* or *C*. *quinquefasciatus* will likely be toxic to both species and that this toxicity is related to similarity in the pharmacological response of the DOP2 receptors. These leads may be more broadly effective against mosquito larvae, as amitriptyline also has been reported to have larvicidal properties against *An*. *gambiae* [21].

In addition to mortality, sublethal effects of antagonists on larval behavior and development were noted throughout the course of this study. Larvae were "debilitated" (i.e., slower and less vigorous in their response to shaking of plates or touch with a sterile probe compared to control larvae) at high concentrations (400–200 μM) of all antagonists with the exception of clomipramine. This was observed as early as 1 h after exposure in *C*. *quinquefasciatus* in response to 400 μM chlorprothixene, methiothepin, or chlorpromazine and 2 h after exposure to chlorprothixene in *Ae*. *aegypti*. Molting to the L4 stage was common for control and treated larvae throughout the experiment, yet the exuviae frequently remained attached to treated larvae, and in particular to debilitated individuals (S5 Fig). Disruption of dopaminergic signaling may partially explain the debilitated phenotype and the difficulty of larvae to completely free themselves from their exuviae as dopamine has a role in locomotion [18, 19]. In addition, a "shortened" phenotype where larval length was reduced (S5 Fig) was noted in some treated larvae (with the exception of amperozide, asenapine, and mianserin), typically occurring within 24 to 48 h after exposure and observed in both L3 and L4 stages. The link to dopamine signaling with this phenotype is less clear and may relate to as yet uncharacterized functions of the DOP2 receptor, or impacts on other receptor types (see below). Further quantitative studies tracking antagonist effects throughout larval development could provide a more comprehensive picture of their impact, and a closer focus on molting would be particularly interesting given the role of dopamine in this process [20].

While the DAR antagonists investigated herein hold promise for rational insecticide design, the mode of action responsible for *in vivo* toxicity remains uncharacterized. The remarkable correlation between *in vitro* data and *in vivo* larval and adult data, confirmed by linear regression [15], and supported in the current study with *C*. *quinquefasciatus*, provides evidence for activity of these compounds at *Cq*DOP2 and *Aa*DOP2 *in vivo*. Attempts to analyze receptor-antagonist interaction *in vivo* via rescue of amitriptyline treated adult mosquitoes with co-injection of dopamine by our group and others [21] have not proven successful to date. Several studies suggest that some of the DAR antagonists employed in the current study may have activity at other biogenic amine receptors that could result in a more complex pharmacological response *in vivo*. For example, an interaction of methiothepin with serotonin receptors expressed in the Malpighian tubules of *Ae*. *aegypti* has been suggested by several studies [36, 37]. Mianserin and chlorpromazine are β-adrenergic-like octopamine receptor antagonists in the moth *Chilo suppressalis* [38] and cis-(Z)-flupenthixol is an antagonist for an α-adrenergic-like octopamine receptor in *Ap*. *mellifera* [39]. Amperozide and asenapine have not, to our knowledge, been used in studies on insects and further investigation of these antagonists against a range of biogenic-amine binding invertebrate GPCRs may yield insights into mode-of-action. An insecticidal discovery approach that disrupts multiple arthropod receptors as has been proposed in human pharmaceutics, *i*.*e*., the magic shotgun versus the magic bullet approach [40], could be an attractive alternative to receptor-specific chemistries.

Improvements in potency and toxicity are typically achieved via medicinal and product formulation chemistry, respectively. The antagonists tested here were unformulated for delivery. Thus degradation, metabolic detoxification by insect enzymes as well as differences in penetration of the cuticle, gut lining, or neural tissue may limit bioavailability. Many of the principles and approaches used in human pharmaceutical research to understand the absorption, distribution, metabolism and excretion (ADME) of molecules could have application for DAR-antagonist insecticide discovery. Improvement in compound delivery with the addition of carriers, synergists or through the use of analogs derived from these chemistries will ideally result in compounds capable of inducing toxicity at nM to pM concentration, a desired target range for use of pesticides in the field.

The PIDP is a target-based pipeline for discovery of new mode-of-action insecticides (Fig. 1) with initial proof of concept provided by studies of the D_1_-like DAR, *Aa*DOP2 [11, 13, 15]. Here we show that the pharmacological properties of the orthologous target, *Cq*DOP2, from a second mosquito vector of NTDs, *C*. *quinquefasciatus*, largely mirror those of *Aa*DOP2, and that the toxicity of *Aa*DOP2-active antagonist leads extends to the larvae of this species. This study brings the number of pharmacologically characterized vector DOP2 DARs in the PIDP to five: *Cq*DOP2 (this study), *Aa*DOP1 and 2 [11], *Is*DOP1 and 2 [14, 22] and extends the activity of pipeline chemistries to a second taxonomic group of vectors in the subfamily Culicinae. Three DOP2 targets, *Ag*DOP2, *Pp*DOP2, and *Gm*DOP2 from the dipteran vectors of malaria, leishmaniasis, and African Trypanosomiasis, and proposed counter-screens against the honeybee DOP2 DAR will enable expansion in the scope and insecticide discovery potential of the PIDP. Larval mosquito assays offer a relatively inexpensive, high-throughput tool to narrow down chemical leads for evaluation in more labor-intensive assays for activity against adult vectors. Combined, these pipeline components enable the discovery of taxon-selective chemistries and the early elimination of molecules with undesirable environmental impacts. While the present study focused on D_1_-like DARs, in theory the PIDP could be applied to discovery of agonists, and inhibitors of D_2_-like and other biogenic amine-binding receptors in the search for GPCR-active, novel insecticides.

### Summary and future work

This study provides evidence for *Cq*DOP2 as a D_1_-like DAR and supports an orthologous relationship to *Aa*DOP2. Within the mosquito subfamily Culicinae, tribes Aedini (including *Ae*. *aegypti*) and Culicini (including *C*. *quinquefasciatus*) are thought to have diverged in the early Cretaceous, an estimated 127 to 158 million years ago [41], yet the degree of sequence and pharmacological similarity, coupled with the detection of transcripts in all life stages, suggests that *Cq*DOP2 and *Aa*DOP2 play a conserved role in mosquito neurological processes and may explain the similar toxic effects of DAR antagonists to the larvae of *C*. *quinquefasciatus* and *Ae*. *aegypti*. These findings are an important step towards understanding the relationship between sequence similarity, DAR pharmacology *in vitro* and antagonist toxicity *in vivo*. Future mutagenesis and modeling work with these receptors may reveal key DAR structural features required for activity that may be used to predict the pharmacology of orthologous targets. This information could be used to assess the value of de-orphanization and development of additional orthologous targets, which represents a considerable time and cost investment. DAR antagonist toxicity to *C*. *quinquefasciatus* suggests these molecules may have activity against other culicine species and possibly other arthropod vectors. The three additional DOP2 targets, *Ag*DOP2, *Pp*DOP2 and *Gm*DOP2, identified in this study (Figs. 1, S2) provide a powerful research tool to investigate DAR antagonist potency to other dipteran vectors and the pharmacological consequences of divergence in amino acid sequences in these species. Importantly, our finding that *Cq*DOP2 and *Aa*DOP2 are pharmacologically distinct from the human D_1_-like DAR, hD_1_, and exhibit a 35 to 227-fold range difference in response to select antagonists suggests that differences between mosquito and human DARs can be exploited for development of mosquito-selective compounds with low mammalian toxicity.

The DAR antagonists tested in this study are tricyclic amines, with the exception of amperozide, a diphenylbutylpiperazine. Modeling of mosquito receptors may allow prediction of ligand binding sites and facilitate directed searches for small molecule inhibitors, potentially revealing new classes of DAR antagonists selective for mosquitoes. Agonists also have potential to disrupt *Aa*DOP2 signaling [13] and should be examined against *Cq*DOP2 and the DOP2 DAR targets of other dipteran vectors identified in this study. The rational, target-based insecticide discovery approach taken in this study provides further evidence that investment in small molecule antagonists of invertebrate GPCRs may deliver much needed novel mode-of-action products to the vector control market.

## Supporting Information

S1 Fig Detection of *Cq*DOP2 transcripts in *C*. *quinquefasciatus* life stages by end-point RT-PCR. Abbreviations: E, egg; L, L4 larva; P, pupa; AF, adult female; AM, adult male.
(TIF)

S2 Fig Alignment of DOP2 amino acid sequences from selected dipteran vectors of NTDs. Black and gray highlighted areas indicate identical and conserved residues as designated by ClustalW [29]: black = identical residues; dark gray = strongly similar residues; light gray = weakly similar residues (for amino acid similarity groups, see: http://www.clustal.org/download/clustalx_help.html). Color coding indicates conserved structural features. Orange = residues required for receptor activation; Blue = biogenic amine interaction sites; Green = putative protein kinase A/C phosphorylation sites; Yellow = putative palmitolyation sites. Putative transmembrane (TM) domains I-VII are indicated as a line above the alignment. NCBI accession numbers of species indicated are as follows: *Culex quinquefasciatus* DOP2 = KM262648; *Aedes aegypti* DOP2 = JN043503; *Anopheles gambiae* DOP2 = ABKP02003382 and ABKP02020596; *Phlebotomus papatasi* DOP2 = AJVK01013962 and AJVK01013961; *Glossina morsitans* DOP2 = CCAG010002977. Sequences were assembled from multiple scaffolds for *An*. *gambiae* and *P*. *papatasi* in order to obtain complete sequences including all three putative exons.
(TIF)

S3 Fig Concentration response curves for *C*. *quinquefasciatus* (○) and *Ae*. *aegypti* (●) showing percent larval mortality at 24 h post exposure to DOP2 antagonists. Each data point represents mean ± SEM (n ≥ 3 independent experiments).
(TIFF)

S4 Fig Concentration response curves for *C*. *quinquefasciatus* (○) and *Ae*. *aegypti* (●) showing percent larval mortality at 48 h post exposure to DOP2 antagonists. Each data point represents mean ± SEM (n ≥ 3 independent experiments).
(TIFF)

S5 Fig Images of sublethal phenotypes observed in *C*. *quinquefasciatus* and *Ae*. *aegypti* larvae following exposure to DAR antagonists. Representative examples of (A) normal (water only control, 72 h exposure) (B) attached exuvia (arrow) (50 μM chlorprothixene, 24 h exposure) and (C) shortened (100 μM chlorpromazine, 72 h exposure) phenotypes in L4 *C*. *quinquefasciatus* and (D) normal (water only control, 72 h exposure), (E) attached exuvia (arrow) (400 μM chlorpromazine, 72 h exposure), and (F) shortened (50 μM methiothepin, 72 h exposure) phenotypes in L4 *Ae*. *aegypti*.
(TIF)
