# Supplementary figures and images for: Dopamine Receptor Antagonists as New Mode-of-Action Insecticide Leads for Control of Aedes and Culex Mosquito Vectors
Source: PLoS Negl Trop Dis. 2015 Mar 20;9(3):e0003515. doi: 10.1371/journal.pntd.0003515 (PMC4368516; doi:10.1371/journal.pntd.0003515)

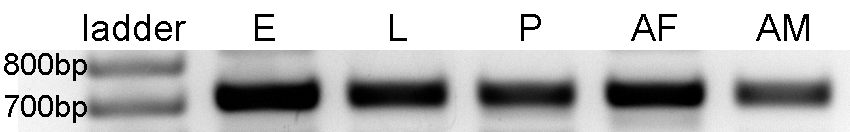

Supplement: S1 Fig — Abbreviations: E, egg; L, L4 larva; P, pupa; AF, adult female; AM, adult male. (TIF) [file pntd.0003515.s001.tif]

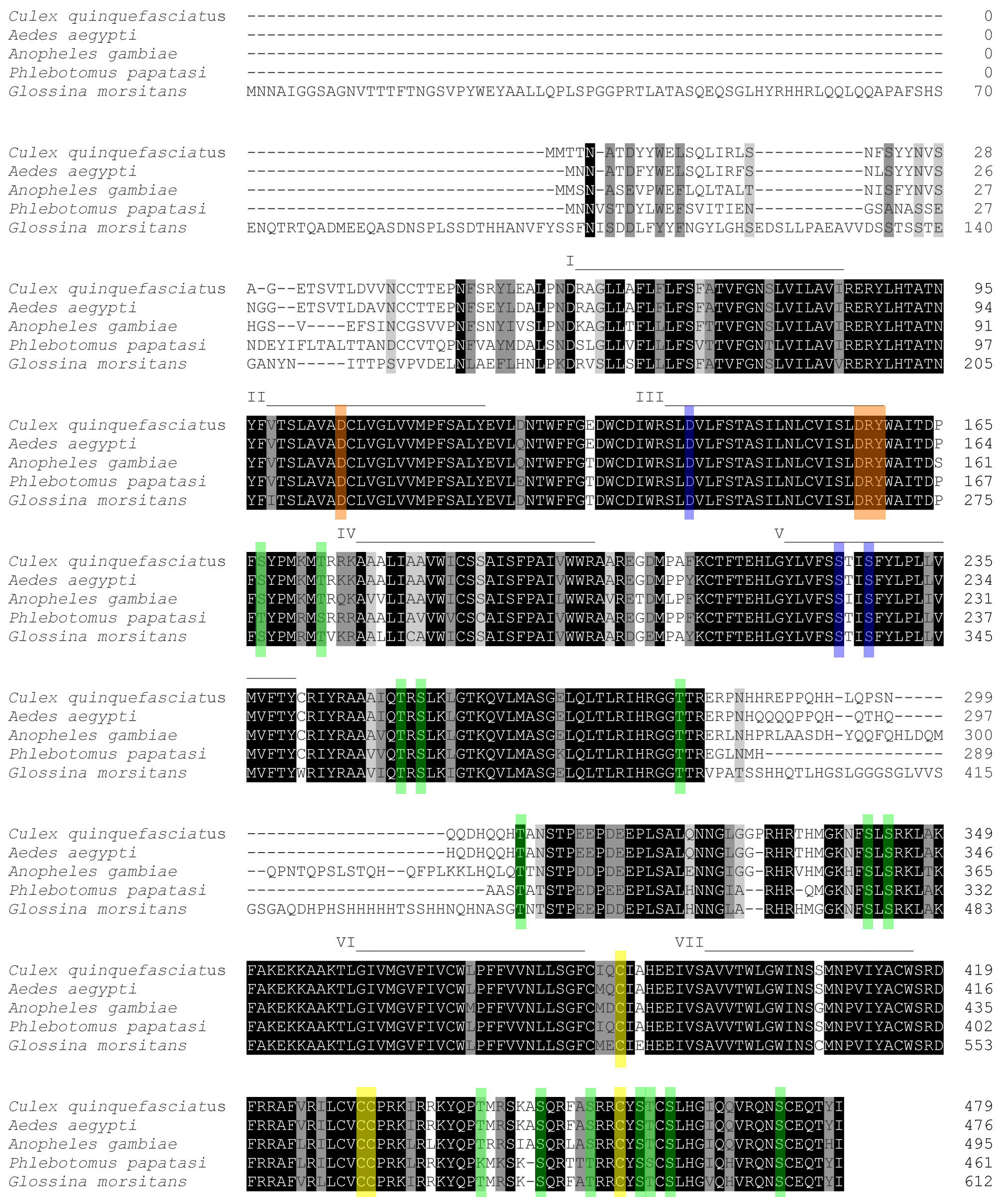

Supplement: S2 Fig — Black and gray highlighted areas indicate identical and conserved residues as designated by ClustalW [29]: black = identical residues; dark gray = strongly similar residues; light gray = weakly similar residues (for amino acid similarity groups, see: http://www.clustal.org/download/clustalx_help.html). Color coding indicates conserved structural features. Orange = residues required for receptor activation; Blue = biogenic amine interaction sites; Green = putative protein kinase A/C phosphorylation sites; Yellow = putative palmitolyation sites. Putative transmembrane (TM) domains I-VII are indicated as a line above the alignment. NCBI accession numbers of species indicated are as follows: Culex quinquefasciatus DOP2 = KM262648; Aedes aegypti DOP2 = JN043503; Anopheles gambiae DOP2 = ABKP02003382 and ABKP02020596; Phlebotomus papatasi DOP2 = AJVK01013962 and AJVK01013961; Glossina morsitans DOP2 = CCAG010002977. Sequences were assembled from multiple scaffolds for An. gambiae and P. papatasi in order to obtain complete sequences including all three putative exons. (TIF) [file pntd.0003515.s002.tif]

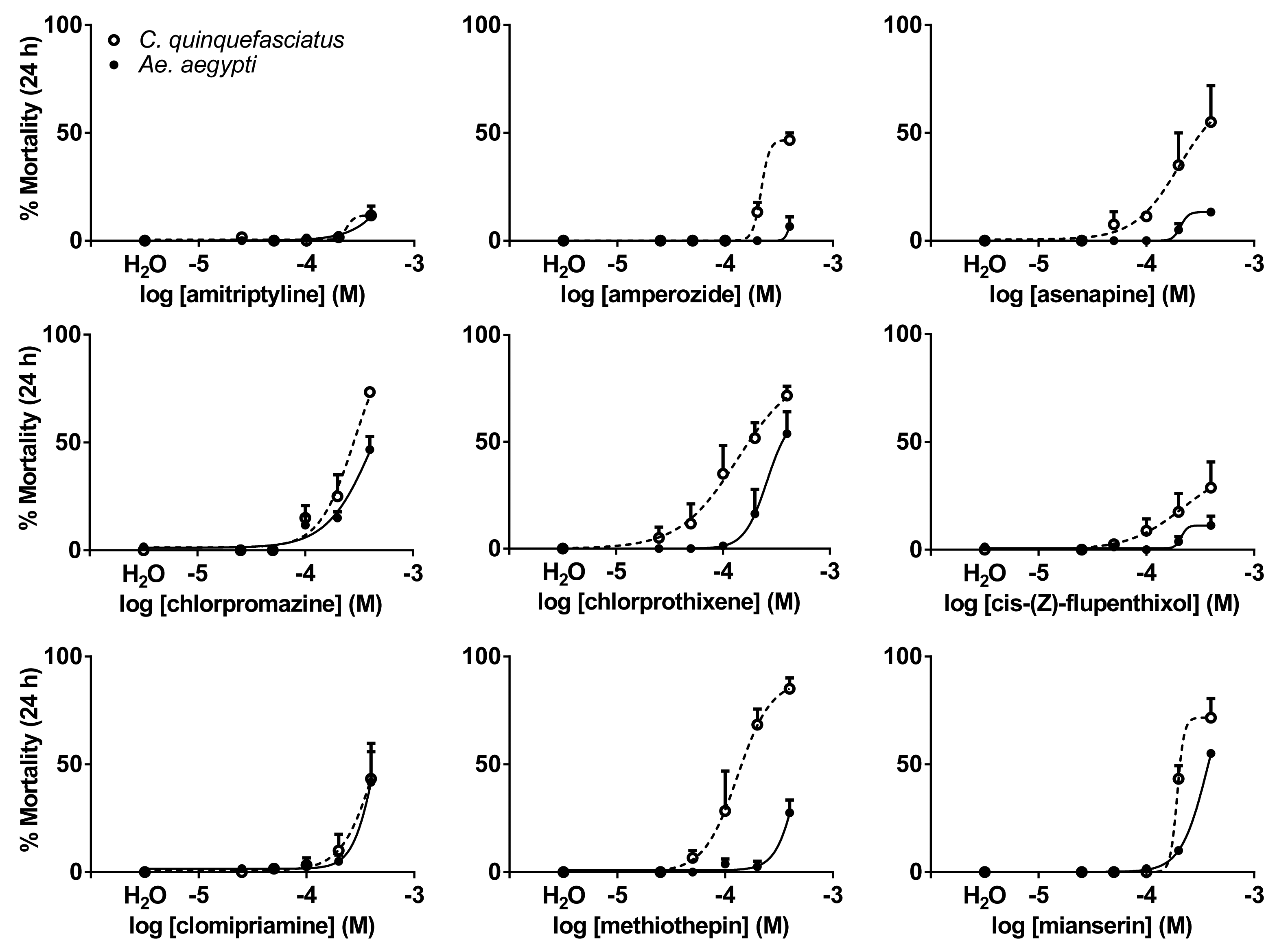

Supplement: S3 Fig — Each data point represents mean ± SEM (n ≥ 3 independent experiments). (TIFF) [file pntd.0003515.s003.tiff]

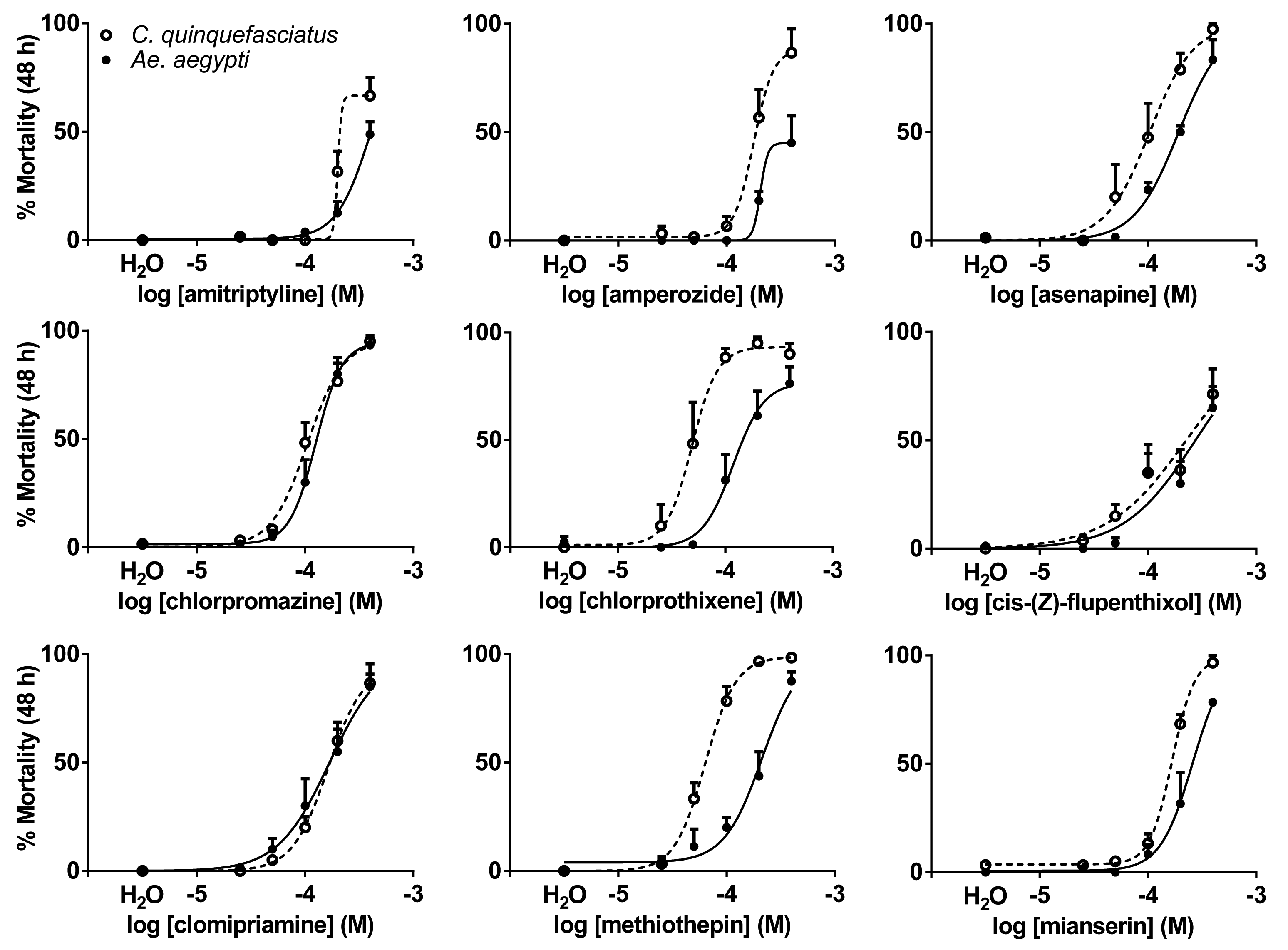

Supplement: S4 Fig — Each data point represents mean ± SEM (n ≥ 3 independent experiments). (TIFF) [file pntd.0003515.s004.tiff]

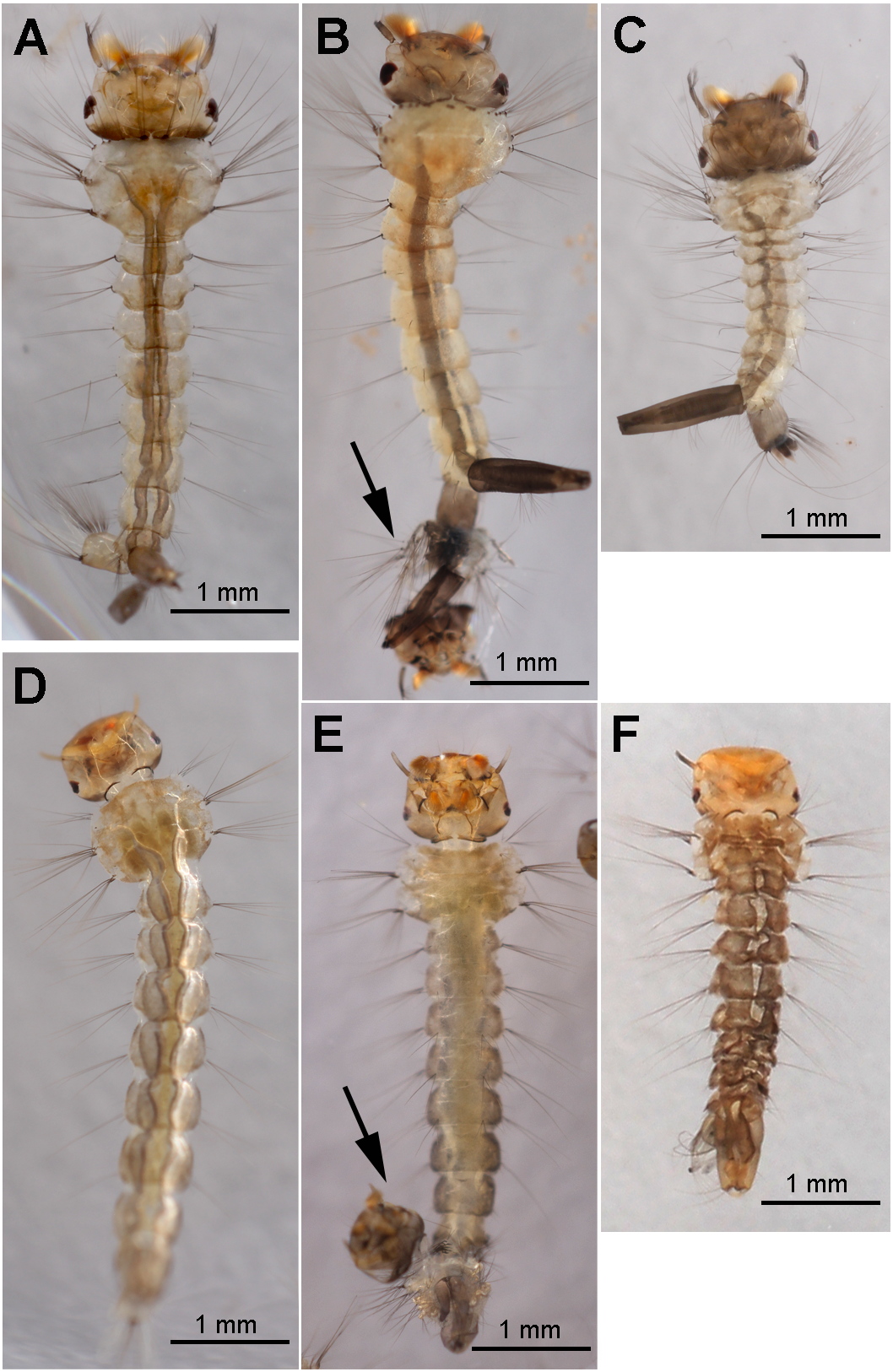

Supplement: S5 Fig — Representative examples of (A) normal (water only control, 72 h exposure) (B) attached exuvia (arrow) (50 μM chlorprothixene, 24 h exposure) and (C) shortened (100 μM chlorpromazine, 72 h exposure) phenotypes in L4 C. quinquefasciatus and (D) normal (water only control, 72 h exposure), (E) attached exuvia (arrow) (400 μM chlorpromazine, 72 h exposure), and (F) shortened (50 μM methiothepin, 72 h exposure) phenotypes in L4 Ae. aegypti. (TIF) [file pntd.0003515.s005.tif]
